# Optical coherence tomography angiography analysis of changes in the retina and the choroid after haemodialysis

**DOI:** 10.1038/s41598-018-35562-6

**Published:** 2018-11-21

**Authors:** Yong Un Shin, Dong Eik Lee, Min Ho Kang, Mincheol Seong, Joo-Hark Yi, Sang-Woong Han, Heeyoon Cho

**Affiliations:** 1 Department of Ophthalmology, Hanyang University College of Medicine, Seoul, Korea; 2 Department of Ophthalmology, Seoul National University College of Medicine, Seoul, Korea; 3 Division of Nephrology, Department of Internal Medicine, Hanyang University College of Medicine, Seoul, Korea

## Abstract

The purpose of this study is to evaluate the effect of haemodialysis on perfused vessel density, choroidal thickness (CT), and retinal thickness in end-stage renal disease (ESRD) using swept-source optical coherence tomography angiography (SS-OCTA). We studied twenty-nine eyes of 29 ESRD patients by ophthalmologic examination and SS-OCTA before and after haemodialysis. The colour-coded perfusion density maps were generated and perfused vessel density was calculated. Changes in systemic and other ocular parameters such as retinal and choroidal thickness were measured and analysed. Total perfused vessel density decreased significantly after haemodialysis in the choriocapillaris; it was not significantly different in the superficial capillary plexus (SCP) and the deep capillary plexus (DCP). Total CT decreased significantly, but total retinal thickness was not significantly different. There was no significant correlation between choriocapillaris perfused vessel density and CT. The reduction in choriocapillaris perfused vessel density correlated with the decrease in systolic and mean arterial blood pressures. The decrease in CT correlated with the ultrafiltration volume. There were no significant systemic and ocular factors affecting change in retinal thickness and perfused vessel density of SCP and DCP. This is the first study to assess the effect of haemodialysis on blood flow changes using SS-OCTA; changes may be more prominent in the choroidal compared to the retinal layer.

## Introduction

End stage renal disease (ESRD) causes systemic accumulation of body fluid. The primary goal of haemodialysis is to maintain the excretory function of the kidneys. During haemodialysis, ultrafiltration reduces the levels of excess water and uremic substances and corrects the fluid balance. Haemodialysis can cause fluctuations in the haemodynamic status, including hypotension, and disturbances in the retrobulbar circulation have been demonstrated^1–5^.

In previous studies, the choroidal and retinal perfusion status after haemodialysis has been assessed using fundus fluorescein angiography (FFA), indocyanine green angiography (ICGA), and ultrasonography (USG)^4,6–8^. However, such traditional imaging modalities are insufficient for quantitative assessment^9^. Although FFA and ICGA are the gold standards for retinal and choroidal vasculature evaluation, small retinal vessels can be masked by subsequent hyperfluorescence, and systemic side effects may occur due to intravenous contrast^10–12^. Thus, most recent studies have primarily investigated the effect of haemodialysis on the choroidal and retinal thickness using optical coherence tomography (OCT). Jung *et al*.^13^ reported an increase in the choroidal thickness (CT) after haemodialysis, but other studies have reported a decrease^14,15^. Ulas *et al*.^14^ reported no change, but Theodossiadis *et al*.^16^ demonstrated a decrease in the retinal thickness after haemodialysis. Because of these conflicting reports, the effect of haemodialysis on the retina and choroid is still unclear.

Commercially available optical coherence tomography angiography (OCTA) is a novel and non-invasive imaging technique that uses spectral-domain optical coherence tomography (SD-OCT) or swept-source optical coherence tomography (SS-OCT) for the 3D visualisation of the choroid and retinal microcirculation. Since OCTA provides detailed assessment of the retinal and choroidal vasculature by detecting the motion of erythrocytes and visualising blood flow using serial OCT B-scans, it is uniquely suited for assessing the layer-specific perfusion status of the macula^17–20^.

Despite the strengths of OCTA, only one study has assessed the vascular changes in the retina and choroid using SD-OCTA^21^. Therefore, the present study was designed to evaluate the effect of haemodialysis on the choroid and retina using SS-OCTA in patients with ESRD.

## Methods

### Subjects

This prospective study included 33 eyes of 33 patients with ESRD undergoing haemodialysis at the Dialysis Center of Hanyang Guri Hospital from May 2016 to June 2016. The study design was approved by the institutional review board at Hanyang University and was performed according to the tenets of the Declaration of Helsinki. Informed consent was obtained from all patients.

Patients with ESRD who were undergoing haemodialysis sessions thrice weekly on alternate days at the Dialysis Center of Hanyang University Guri Hospital (Guri, South Korea) were recruited. The subjects received haemodialysis sessions, each lasting 3–4 h, on alternate days, with high-performance dialysers, at a blood flow rate of 250 ml/min and a dialysate flow rate of 500 ml/min as standard.

We included patients with a best-corrected visual acuity (BCVA) exceeding 6/60 and no history of any retinal diseases with the exception of mild non-proliferative diabetic retinopathy (NPDR). Only the right eyes of patients were included and analysed. Patients with a history of glaucoma; an insufficient or excessive axial length (<21 mm or >27 mm); recent history (within 3 months) of vitrectomy surgery, intravitreal injection, or laser treatment; or poor-quality OCTA images (image quality of less than 60 according to the OCTA manufacturer) due to eye movements or media opacities were excluded from the study.

### Measurements

All participants were examined at the first haemodialysis session of the week (Monday or Tuesday). All measurements were carried out next to the dialysis centre. OCTA was performed immediately before and after a single haemodialysis session (from 7 AM to 11 AM) to avoid diurnal variations. The systolic blood pressure (SBP), diastolic blood pressure (DBP), body weight, and ultrafiltration volume were measured within 5 min before and after haemodialysis. A detailed ophthalmologic examination including measurement of intraocular pressure (IOP), BCVA, and anterior segment parameters was performed within 15 min before and after haemodialysis. The IOP was measured using the Tono-pen (Reichert, USA).

**Figure 1:**
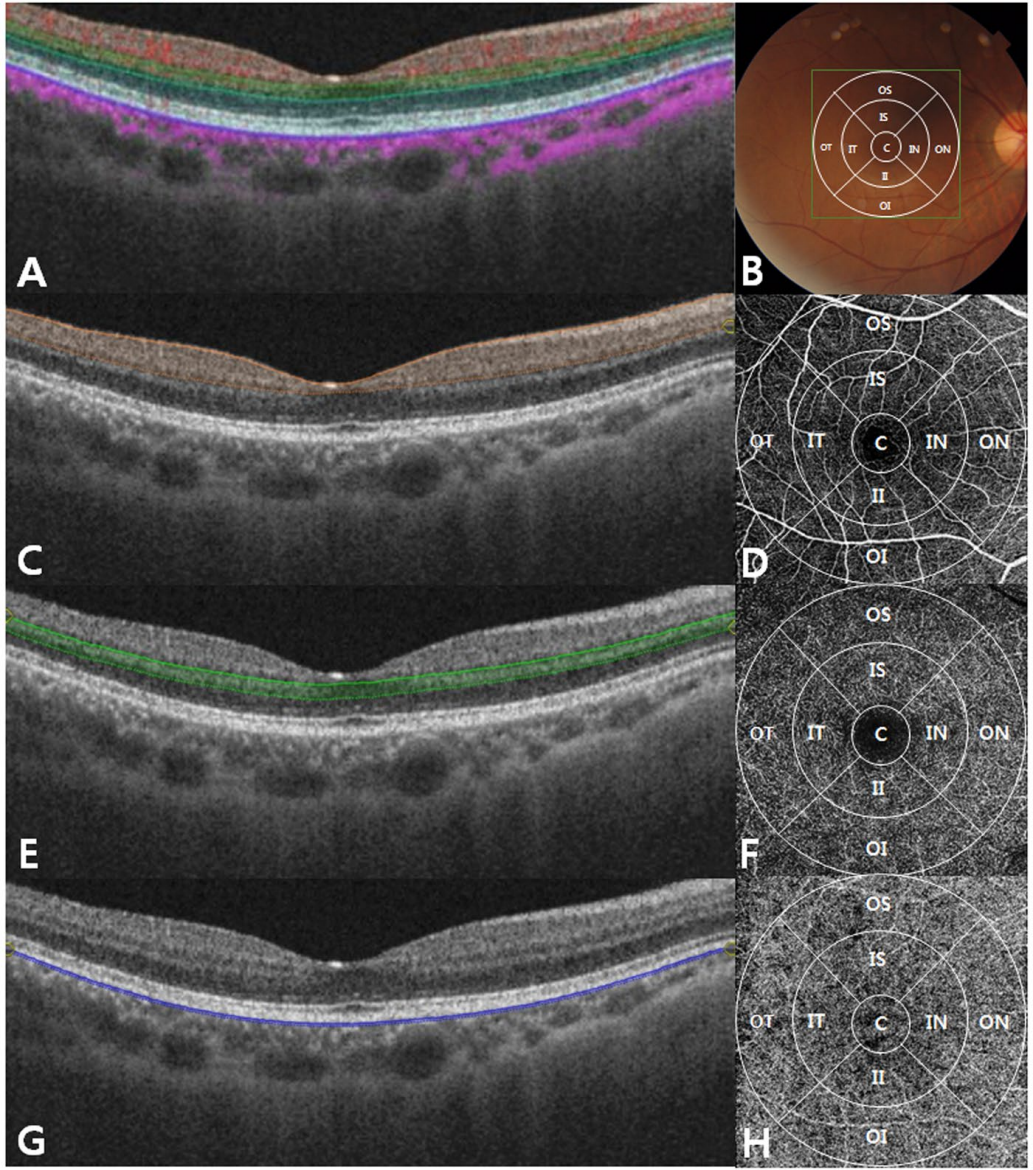
Swept-source optical coherence tomography (OCT) high resolution single scan (**A**), and corresponding fundus imaging with the Early Treatment Diabetic Retinopathy Study (ETDRS) grid (**B**). Single scan swept-source OCT image and corresponding *en face* OCT angiography of the superficial capillary plexus (**C**,**D**), respectively; from 2.6 µm below the internal limiting membrane to 15.6 µm below the interface of the inner plexiform layer and inner nuclear layer (IPL/INL)), the deep capillary plexus (**E**,**F**), respectively; from 15.6 µm below the IPL/INL to 70.2 µm below the IPL/INL), and the choriocapillaris (**G**,**H**), respectively; from the basement membrane to 10.4 µm below the basement membrane). C = central, OS = outer superior, ON = outer nasal, OI = outer inferior, OT = outer temporal, IS = inner superior, IN = inner nasal, II = inner inferior, IT = inner temporal.

**Figure 2:**
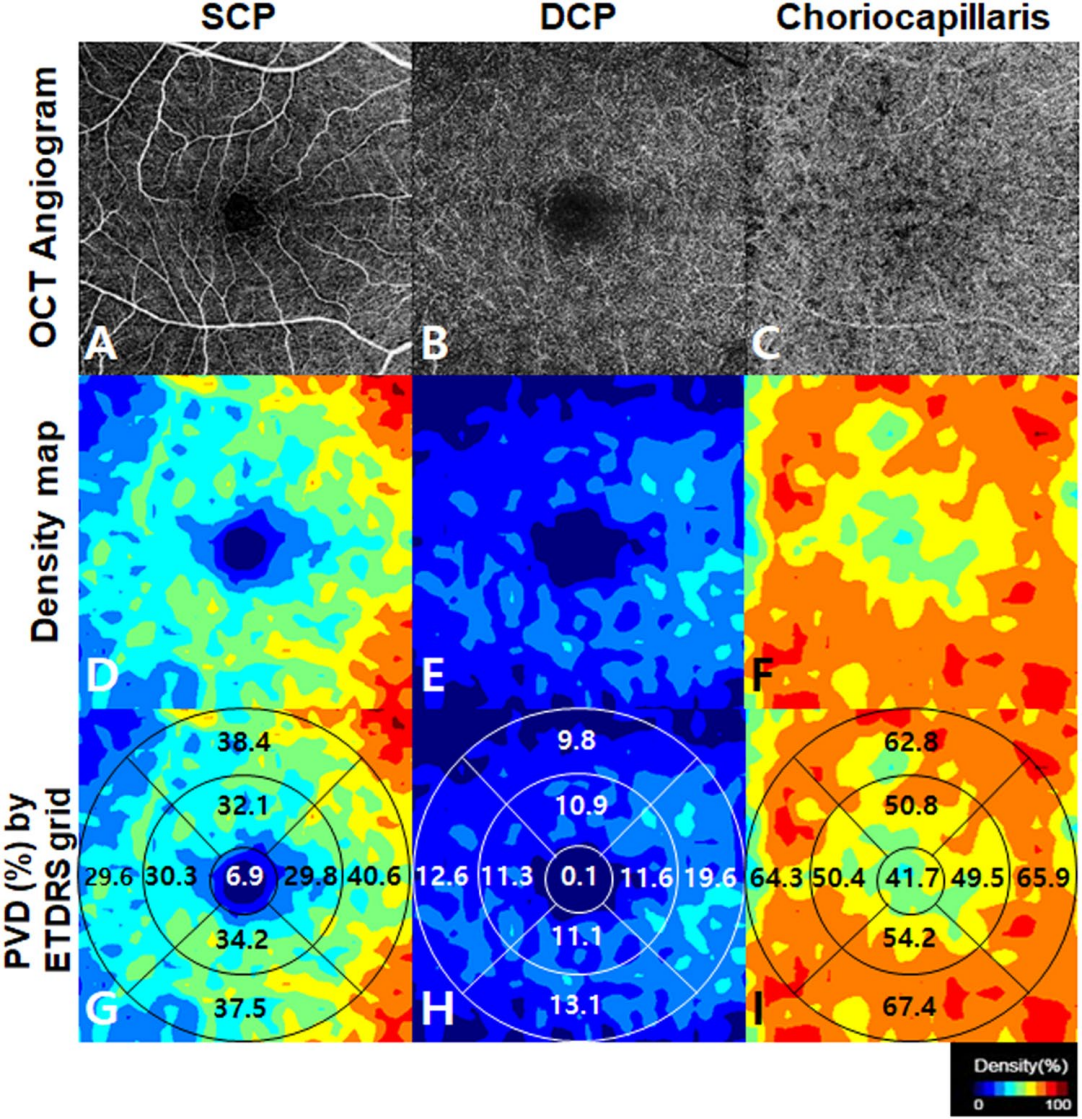
Representative optical coherence tomography angiography (OCTA) images obtained by DRI OCT-1 Atlantis® (Topcon Corporation, Tokyo, Japan). Original grayscale images at the level of superior capillary plexus (**A**), deep capillary plexus (**B**), and choriocapillaris (**C**) were obtained using a 6 × 6 mm protocol centred on the fovea. Colour-coded perfusion density maps at each layer (**D**–**F**) were automatically generated. Numerical information about perfused vessel density (PVD) of each subfield divided into 9 zone Early Treatment Diabetic Retinopathy Study (ETDRS) grid was calculated using Matlab® (The MathWorks, Inc., Natick, MA, USA) (**G–I**).

**Figure 3:**
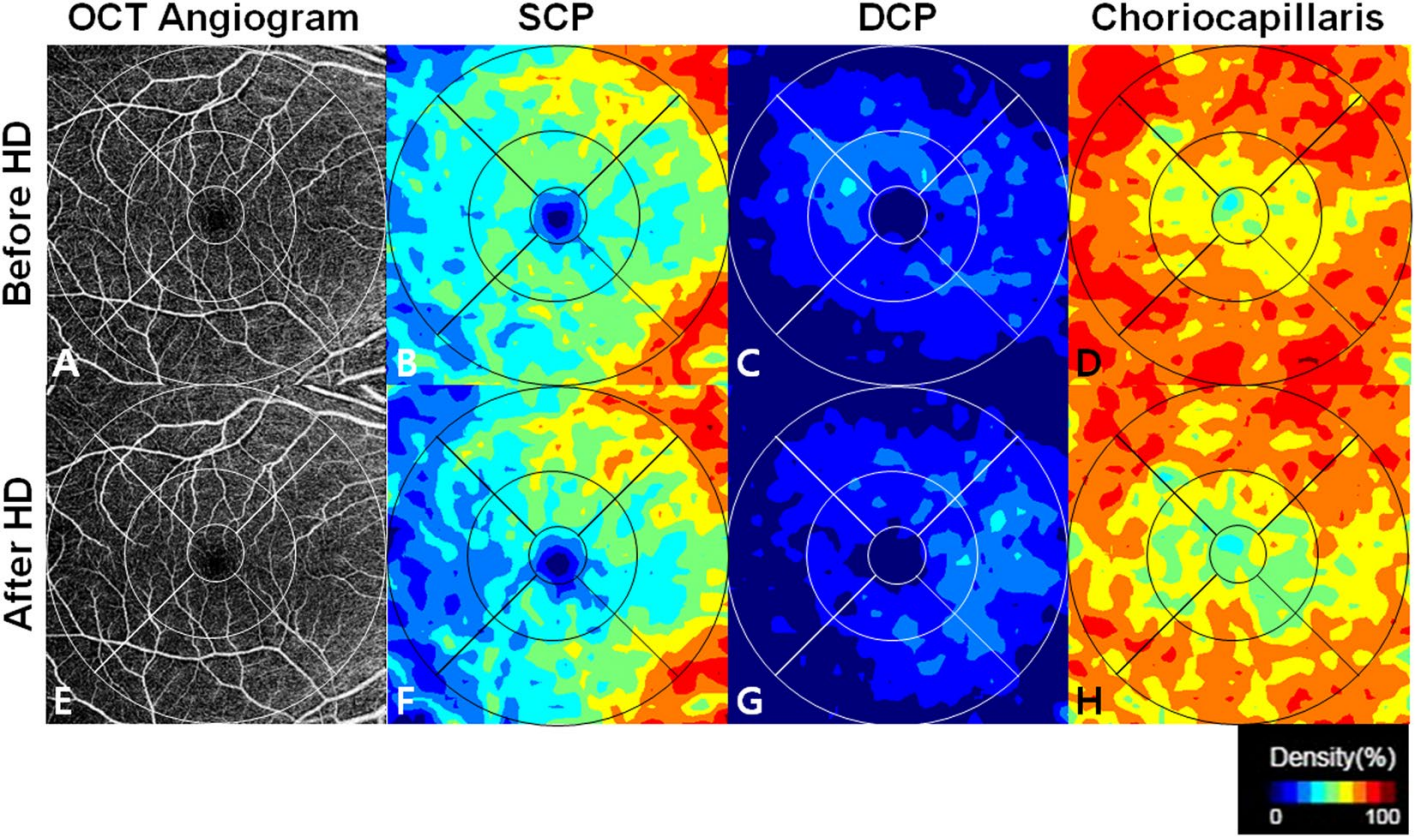
Representative examples of swept-source optical coherence tomography angiography showing changes in perfused vessel density before (**A–D**) and after (**E–H**) haemodialysis. Using these density maps and a colour-scaled bar, a numeric perfusion value (%) is calculated for each layer with Matlab® (The MathWorks, Inc., Natick, MA, USA). The decreased perfused vessel density in all subfields at the choriocapillaris can be appreciated qualitatively and quantitatively with the perfusion density map after haemodialysis. HD = haemodialysis, SCP = superficial capillary plexus, DCP = deep capillary plexus.

OCTA scans were obtained using a DRI OCT-1 Atlantis® scanner (Topcon Corporation, Tokyo, Japan). This system uses a swept source laser with a scanning rate of 100,000 A-scans per s and a longer wavelength of 1050 nm than that of conventional SD-OCT. An active eye tracker was employed to reduce motion and blinking artefacts during OCTA. All eyes were scanned using a 6 × 6 mm protocol centred on the fovea. We used the “remove line noise” option to correct motion artefacts. The “project artefact removal” option was only activated to review the outer retinal layer. The OCTA scans were automatically segmented by the included software into four en-face slabs; (1) the superficial capillary plexus (SCP): from 2.6 µm below the internal limiting membrane to 15.6 µm below the interface of the inner plexiform layer and inner nuclear layer (IPL/INL), (2) the deep capillary plexus (DCP): from 15.6 µm below the IPL/INL to 70.2 µm below the IPL/INL, (3) the outer retinal slab: from 70.2 µm below the IPL/INL to Bruch’s membrane (BM), and (4) the choriocapillaris: from BM to 10.4 µm below BM. OCTA provides non-invasive, high-resolution volumetric blood flow images by motion contrast imaging of erythrocytes^18,22^ (Fig. 1). This technique allows OCTA instruments to automatically generate colour-coded perfusion density maps of the choroidal and retinal circulation in a few seconds^22–25^. In the colour map, there is a colour-scaled bar on which dark red represents a high density of perfused vessels, dark blue represents a low density, and a spectrum of green to yellow represents intermediate densities. The perfusion density map can be interpreted numerically using the corresponding colour scaled information. The macular area of the image was divided into nine zone Early Treatment Diabetic Retinopathy Study (ETDRS) grids. The mean perfused vessel density of each subfield was measured by calculating the average of the colour information of the area using Matlab® (The MathWorks, Inc., Natick, MA, USA). This analysis was performed on three layers of the SCP, DCP, and choriocapillaris in the 6 × 6 mm scans of each subject (Figs 2, 3).

The CT and retinal thickness at the macula were also measured in all subjects. The CT and retinal thickness in nine subfields of the ETDRS grid were obtained using the same DRI OCT-1 Atlantis® device (Topcon Corporation, Tokyo, Japan) used to measure perfused vessel density. The CT and retinal thickness (inner limiting membrane to the outer border of the retinal pigment epithelium) were automatically calculated using a macular 3D scan (512 × 256 A scans/0.8 s) of the built-in software in each subfield.

Two retinal specialists (YUS and HC) reviewed all OCT and OCTA images before and after automated analysis and corrected all segmentation errors. Data that were difficult to analyse due to severe artefacts were excluded.

### Statistical analysis

SPSS version 22.0 (SPSS, Inc., Chicago, IL, USA) was used for all statistical analyses. The Wilcoxon signed-rank test was used to compare IOP, axial length, CT, retinal thickness, and perfused vessel density before and after haemodialysis. Correlations between the measurements were analysed by Pearson’s correlation test. Regression analysis was performed to identify factors related to the changes in the choriocapillaris perfused vessel density and the CT. Subgroup analysis was performed between patients with or without diabetes mellitus (DM) using the Mann-Whitney U test. A *P*-value of <0.05 was considered statistically significant.

## Results

### Baseline characteristics

We studied 29 eyes of 29 patients (12 males, 17 females; age, 39–76 years; mean age, 55.6 ± 9.9 years) who met the inclusion criteria and were enrolled in the study. We excluded four patients: one due to macular oedema (one eye) and three due to low-quality SS-OCT images (three eyes). The aetiology of ESRD included DM (n = 15), hypertensive nephrosclerosis (n = 6), immunoglobulin A nephropathy (n = 3), chronic glomerulosclerosis (n = 2), and unknown origin (n = 3). The mean body weight decreased from 62.4 ± 11.4 kg to 59.7 ± 11.4 kg after haemodialysis (Wilcoxon signed-rank test, *P* < 0.001). The mean SBP decreased from 152.8 ± 32.5 mmHg to 142.7 ± 29.5 mmHg after haemodialysis (Wilcoxon signed-rank test, *P* = 0.014). The baseline characteristics of the patients are presented in Table 1.

**Table 1:** Baseline patient characteristics before and after haemodialysis.

	Before haemodialysis	After haemodialysis	P*
n	29		
Age, years	55.6 ± 9.9		
Gender (male/female)	12/17		
DM/Non-DM	15/14		
HTN/Non-HTN	20/9		
Duration of HD, months	69.3 ± 47.8		
Ultrafiltration volume, L	2.9 ± 0.8		
Body weight, kg	62.4 ± 11.4	59.7 ± 11.4	**<0.001** ^**†**^
SBP, mmHg	152.8 ± 32.5	142.7 ± 29.5	**0.014** ^**†**^
DBP, mmHg	77.5 ± 11.9	79.8 ± 11.7	0.369
MABP, mmHg	102.6 ± 12.7	100.8 ± 15.3	0.476
IOP, mmHg	17.4 ± 3.4	16.6 ± 3.1	0.088

DM, diabetes mellitus; HTN, hypertension; HD, haemodialysis; SBP, systemic blood pressure; DBP, diastolic blood pressure; MABP, mean arterial blood pressure; IOP, intraocular pressure. *Wilcoxon signed-rank test. Bold^†^ - Statistically significant difference (*P* < 0.05).

### Effect of haemodialysis on perfused vessel density

The mean total perfused vessel density in the choriocapillaris decreased from 46.2 ± 11.3% to 43.3 ± 10.7% (Wilcoxon signed-rank test, *P* < 0.001). The perfused vessel density in the choriocapillaris decreased significantly in all subfields with the exception of the inner inferior subfield Wilcoxon signed-rank test, *P* = 0.153). In contrast, the perfused vessel density in the SCP and DCP did not change significantly in the vast majority of the subfields. The changes in the perfused vessel density of the macula after haemodialysis are listed in Table 2.

**Table 2:** Changes in perfused vessel density at the level of the superficial capillary plexus, deep capillary plexus and choriocapillaris after haemodialysis.

	SCP			DCP			Choriocapillaris		
	Before HD	After HD	P*	Before HD	After HD	P*	Before HD	After HD	P*
Total	21.7 ± 6.3	21.9 ± 5.7	0.772	8.5 ± 5.7	8.9 ± 4.8	0.512	46.2 ± 11.3	43.3 ± 10.7	**<0.001** ^†^
C	5.2 ± 5.6	5.3 ± 3.8	0.917	2 ± 3.2	1.5 ± 1.8	0.291	37.5 ± 14.3	34.7 ± 14.1	**0.007** ^†^
OS	32.3 ± 6.3	27.9 ± 7.1	**0.001** ^†^	10.2 ± 6.1	7.2 ± 5.3	**0.015** ^†^	51.4 ± 10.4	47.2 ± 9.7	**<0.001** ^†^
ON	31.6 ± 8.2	30.5 ± 8.7	0.546	11 ± 8.3	11.9 ± 8	0.525	50.1 ± 11.3	47.1 ± 9.8	**0.003** ^†^
OI	25.3 ± 7.4	27.8 ± 6.1	**0.011** ^†^	7.2 ± 6.7	9.7 ± 5.8	**0.028** ^†^	50.5 ± 11.5	48.1 ± 10.6	**0.002** ^†^
OT	20 ± 7.1	20.6 ± 5.3	0.621	8.4 ± 6	9 ± 5.3	0.610	50.3 ± 11.2	48.3 ± 10.1	**0.032** ^†^
IS	24.9 ± 8.2	24.9 ± 8.8	0.988	10.5 ± 8.1	10.5 ± 7.4	0.989	45.3 ± 12.1	41.1 ± 12.1	**<0.001** ^†^
IN	18.8 ± 10	19.5 ± 10	0.572	10.9 ± 9.2	11.4 ± 8.3	0.674	42 ± 13.8	38.9 ± 12.5	**<0.001** ^†^
II	18.5 ± 9.5	20.1 ± 7.7	0.183	8.4 ± 8.3	9.8 ± 5.7	0.260	42.8 ± 13.3	41.5 ± 13	0.153
IT	18.5 ± 8.6	20.5 ± 6.6	**0.048** ^†^	8.3 ± 6.8	9.7 ± 5.4	0.088	45.7 ± 11.1	42.5 ± 11	**0.001** ^†^

SCP, superficial capillary plexus; DCP, deep capillary plexus; HD, haemodialysis; C, central; OS, outer superior; ON: outer nasal; OI, outer inferior OT, outer temporal; IS, inner superior; IN, inner nasal; II, inner inferior; IT, inner temporal. *Wilcoxon signed-rank test. Bold^†^ - Statistically significant difference (*P* < 0.05).

### Changes in the retinal and choroidal thickness after haemodialysis

The mean CT decreased significantly in all subfields, including the total and the central areas (−14.4 ± 10.2 µm and −15.7 ± 18.3 µm), but the retinal thickness did not significantly change in any subfield after haemodialysis. The detailed values of the choroidal and retinal thickness in each subfield are presented in Table 3.

**Table 3:** Changes in choroidal thickness and retinal thickness after haemodialysis.

	CT			RT		
	Before HD	After HD	P*	Before HD	After HD	P*
Total	209.6 ± 64.6	195.2 ± 64.1	**<0.001** ^**†**^	267.3 ± 15.5	267.2 ± 15.5	0.866
C	214.8 ± 80.9	199.1 ± 83.2	**<0.001** ^**†**^	235.7 ± 40.6	235.4 ± 41.5	0.759
OS	219.4 ± 70.4	208 ± 67.6	**0.002** ^**†**^	255.3 ± 23.5	255.7 ± 22.8	0.596
ON	182.3 ± 58.6	164.6 ± 56.3	**<0.001** ^**†**^	283.4 ± 20.9	283.5 ± 20.6	0.969
OI	194.3 ± 68.8	179.9 ± 64.4	**<0.001** ^**†**^	254.4 ± 24.7	255.4 ± 25.4	0.137
OT	207.6 ± 60.7	194.4 ± 61.3	**<0.001** ^**†**^	254.4 ± 23.8	254.5 ± 24.5	0.897
IS	224.3 ± 76.1	210.9 ± 76.6	**0.001** ^**†**^	290.0 ± 17.7	290.1 ± 17.9	0.897
IN	214.5 ± 75.9	196.5 ± 75.7	**<0.001** ^**†**^	282.8 ± 26.7	281.8 ± 25.6	0.153
II	214.4 ± 74.1	199.5 ± 72.3	**<0.001** ^**†**^	276.8 ± 34.2	276.6 ± 33.9	0.744
IT	214.4 ± 68.7	203.4 ± 70.9	**0.003** ^**†**^	272.4 ± 26.3	271.5 ± 24.7	0.187

CT, choroidal thickness; RT, retinal thickness; HD, haemodialysis. C, central; OS, outer superior; ON: outer nasal; OI, outer inferior OT, outer temporal; IS, inner superior; IN, inner nasal; II, inner inferior; IT, inner temporal. *Wilcoxon signed-rank test. Bold^†^ - Statistically significant difference (*P* < 0.05).

### Subgroup analysis between participants with and without diabetes

The participants were divided into two groups (15 eyes with DM and 14 eyes without DM). The parameters, including the systemic and ocular variables, did not significantly differ between the two groups before and after haemodialysis (Mann-Whitney *U* test, all *P* > 0.05). The comparisons between the two groups are listed in Table 4.

**Table 4:** Comparisons of patients with and without diabetes.

	DM	Non-DM	P*
Age, years	56.6 ± 9.7	54.5 ± 10.5	0.694
Gender (male/female)	8/7	4/10	
Duration of HD, months	58.1 ± 47.9	81.3 ± 14.3	0.169
Ultrafiltration Volume, L	2.9 ± 0.7	3.0 ± 0.9	0.810
Changes in Weight loss, kg	2.5 ± 0.8	2.8 ± 0.7	0.303
Changes in SBP, mmHg	−11.3 ± 23.3	−8.6 ± 18.5	0.894
Changes in DBP, mmHg	−0.5 ± 16.4	5.4 ± 10.1	0.114
Changes in MABP, mmHg	−4.1 ± 15.6	0.7 ± 10.9	0.238
Changes in IOP, mmHg	−1.1 ± 2.5	0.2 ± 3.0	0.085
Changes in *c*CVD	−3.5 ± 6.1	2.1 ± 4.2	0.585
Changes in *t*CVD	−3.2 ± 3.7	−2.6 ± 3.7	0.615
Changes in *c*CT	−19.5 ± 13.8	−11.6 ± 21.8	0.498
Changes in *t*CT	−15.9 ± 7.6	−12.8 ± 12.5	0.383
Changes in *c*RT	−0.3 ± 2.1	0.7 ± 3.9	0.809
Changes in *t*RT	−0.3 ± 3.4	0.5 ± 3.4	0.265
Changes in *c*SVD	1.2 ± 2.8	−1.1 ± 5.6	0.198
Changes in *t*SVD	1.0 ± 3.3	−0.6 ± 4.9	0.337
Changes in *c*DVD	−0.2 ± 2.1	−1.0 ± 3.3	0.860
Changes in *t*DVD	0.7 ± 2.1	0.1 ± 4.4	0.485

DM, diabetic mellitus; HD, haemodialysis; SBP, systolic blood pressure; DBP, diastolic blood pressure; MABP, mean arterial blood pressure; IOP, intraocular pressure; *c*, central area; *t*, total area; CVD, perfused vessel density in the choriocapillaris; CT, choroidal thickness; RT, retinal thickness; SVD, perfused vessel density in the superficial capillary plexus; DVD, perfused vessel density in the deep capillary plexus *Mann-Whitney U test.

### Associations between the changes in structural OCT and OCTA

There was no significant correlation between the change in the perfused vessel density in the choriocapillaris and the change in the CT in any subfield including the total (Pearson’s correlation coefficient = 0.05; *P* = 0.781) and the central areas (Pearson correlation coefficient = −0.05; *P* = 0.792). The absence of a correlation between the change in the perfused vessel density in the choriocapillaris and the change in the CT was consistent among the subfields of the diabetic and nondiabetic groups (all *P* > 0.05).

Additionally, there was no significant correlation between the change in the perfused vessel density in the SCP or DCP and the change in the retinal thickness in the majority of subfields including the total and the central areas. The detailed values are presented in Table 5.

**Table 5:** Correlation between the change in retinal thickness and perfused vessel density at the level of superficial capillary plexus and deep capillary plexus in each subfield.

	SCP			DCP		
	All	DM	Non-DM	All	DM	Non-DM
Total	−0.35	−0.35	−0.38	−0.15	−0.09	−0.16
C	−0.27	−0.38	−0.21	0.04	0.01	0.10
OS	−0.03	0.21	−0.03	−0.17	0.13	−0.27
ON	−**0.40**^**†**^	−0.24	−0.50	−**0.49**^**†**^	−**0.64**^**†**^	−0.47
OI	−0.09	−0.10	−0.10	0.16	0.15	0.18
OT	0.06	0.01	−0.11	0.31	0.04	**0.55** ^**†**^
IS	−0.37	−**0.52**^**†**^	−0.24	−0.27	−0.39	−0.15
IN	−0.36	−0.36	−0.40	−0.27	−0.24	−0.33
II	−**0.47**^**†**^	−0.29	−**0.59**^**†**^	−0.18	−0.03	−0.26
IT	0.21	**0.61** ^**†**^	−0.34	0.31	0.37	0.27

SCP, superficial capillary plexus; DCP, deep capillary plexus. C, central; OS, outer superior; ON: outer nasal; OI, outer inferior OT, outer temporal; IS, inner superior; IN, inner nasal; II, inner inferior; IT, inner temporal. Pearson’s correlation test was performed between changes in retinal thickness and perfused vessel density at the level of the SCP and DCP in each subfield. Bold^†^ - Statistically significant difference (*P* < 0.05).

### Associations between the changes in the OCT-parameters and systemic factors

No significant association was observed between the changes in the retinal thickness and systemic factors (all *P* > 0.05). The change in the CT was significantly correlated with the ultrafiltration volume, and could explain 16% of the changes in the CT in the total area (R² = 0.162, *P* = 0.030).

The relationships between the change in the perfused vessel density on OCTA and systemic factors were analysed. Univariate regression analysis revealed no significant associations between the changes in vessel density in the SCP and DCP, and systemic factors, such as the presence of DM or hypertension, changes in SBP, DBP, mean arterial pressure (MAP), and ultrafiltration rate (all *P* > 0.05). Multivariate regression analysis after adjustment for all confounding systemic factors revealed that no factors exhibited significant associations with the change in the inner retinal vessel density (all *P* > 0.05). In contrast, there was a statistically significant correlation between the change in the choriocapillaris perfused vessel density and the change in blood pressure (BP) (Table 6). Stepwise multivariate linear regression analysis revealed that a change in the MAP could explain 26% of the changes in the choriocapillaris perfused vessel density at the central area (R² = 0.256, *P* = 0.005), and changes in the SBP could explain 31% of the changes in the choriocapillaris perfused vessel density in the total area (R² = 0.314, *P* = 0.002) in all patients.

**Table 6:** Correlation between changes in systolic blood pressure, diastolic blood pressure, mean arterial blood pressure, ultrafiltration volume, intraocular pressure, and changes in choriocapillaris perfused vessel density and choroidal thickness.

		Age		Duration of HD		Weight		SBP		DBP		MABP		UF		IOP		Presence of HTN	
		r	*P*	r	*P*	r	*P*	r	*P*	r	*P*	r	*P*	r	*P*	r	*P*	r	*P*
Central CVD	All	0.13	0.247	−0.04	0.413	−0.03	0.444	0.48	**0.005** ^**†**^	0.39	**0.019** ^**†**^	0.51	**0.003** ^**†**^	0.54	0.390	0.08	0.344	−0.16	0.408
	DM	0.11	0.342	0.13	0.324	−0.08	0.385	0.54	**0.020** ^**†**^	0.26	0.172	0.45	**0.046** ^**†**^	0.26	0.172	0.14	0.315	−0.12	0.682
	Non−DM	0.21	0.241	−0.41	0.072	0.13	0.326	0.35	0.107	0.64	**0.007** ^**†**^	0.59	**0.013** ^**†**^	−0.19	0.258	−0.07	0.404	−0.26	0.371
Total CVD	All	0.35	**0.030** ^**†**^	−0.16	0.210	0.31	0.054	0.56	**0.001** ^**†**^	0.27	0.077	0.47	**0.005** ^**†**^	−0.11	0.287	0.36	0.072	−0.20	0.292
	DM	0.38	0.080	0.11	0.346	0.30	0.138	0.68	**0.002** ^**†**^	0.01	0.481	0.35	0.101	0.29	0.148	0.39	0.067	−0.02	0.935
	Non−DM	0.35	0.107	−0.52	**0.030** ^**†**^	0.36	0.101	0.40	0.079	0.71	**0.002** ^**†**^	0.66	**0.005** ^**†**^	−0.44	0.058	0.13	0.326	−0.42	0.139
Central CT	All	−0.10	0.304	−0.23	0.111	−0.09	0.325	−0.09	0.330	0.17	0.184	0.07	0.351	−0.32	**0.046** ^**†**^	0.12	0.266	−0.10	0.619
	DM	−0.12	0.337	0.04	0.440	−0.31	0.133	−0.42	0.060	0.11	0.349	−0.13	0.320	0.16	0.282	−0.01	0.487	−0.26	0.347
	Non−DM	−0.05	0.427	−0.57	**0.017** ^**†**^	0.14	0.317	0.16	0.292	0.19	0.262	0.21	0.240	−0.59	**0.013** ^**†**^	0.11	0.358	−0.01	0.974
Total CT	All	0.02	0.462	−0.25	0.096	0.10	0.305	−0.11	0.286	0.04	0.427	−0.03	0.435	−0.40	**0.015** ^**†**^	0.17	0.188	0.04	0.853
	DM	−0.07	0.398	−0.02	0.472	0.08	0.391	−0.53	**0.022** ^**†**^	−0.19	0.252	−0.39	0.074	−0.06	0.417	−0.08	0.388	0.17	0.545
	Non−DM	0.10	0.363	−0.51	**0.032** ^**†**^	0.18	0.270	0.20	0.251	0.20	0.246	0.24	0.209	−0.59	**0.013** ^**†**^	0.25	0.192	−0.07	0.824

HD, haemodialysis; SBP, systolic blood pressure; DBP, diastolic blood pressure; MABP, mean arterial blood pressure; UF, ultrafiltration volume; IOP, intraocular pressure; CVD, the changes in choriocapillaris perfused vessel density; CT, the changes in choroidal thickness. DM, diabetic mellitus. *Pearson’s correlation (r = Pearson’s correlation coefficient). Bold^†^ - Statistically significant difference (*P* < 0.05).

## Discussion

Although previous reports have examined the effect of haemodialysis on the macula, these studies were limited to measurement of the choroidal and retinal thickness using OCT, and reported variable findings^13–16,26,27^. In the current study, we used SS-OCTA to investigate the change in the perfused vessel density of the macula after haemodialysis, and determined the relationship between the changes in the perfused vessel density, CT, retinal thickness, and other parameters. Haemodialysis induced significant decreases in body weight, SBP, and the choriocapillaris perfused vessel density. The decrease in the choriocapillaris perfused vessel density was correlated with the decrease in BP.

In the recent past, several studies have examined the effect of haemodialysis on CT. Jung *et al*.^13^ reported that the subfoveal CT increased after haemodialysis as measured by SD-OCT without using the enhanced depth imaging (EDI) technique. However, the majority of studies reported that the CT decreased after haemodialysis. Ishibazawa *et al*.^28^ reported that the decrease in CT was associated with fluid removal during haemodialysis, as measured by SD-OCT without EDI. Chang *et al*.^26^ reported that the decrease in CT was correlated with the decrease in body weight, SBP, and serum osmolarity using EDI-OCT. Yang *et al*.^15^ reported that the decrease in CT measured by EDI-OCT was associated with a decrease in body weight. Ulas *et al*.^14^ did not find any variable to be associated with a decrease in the CT, measured using EDI-OCT, after haemodialysis. Several studies have reported that there was no significant change in the retinal thickness after haemodialysis in patients without diabetic macular oedema^14,29^. Theodossiadis *et al*.^16^ demonstrated that the retinal thickness decreased after haemodialysis in patients with DM-induced macular oedema, while there was a less pronounced but significant effect in those without oedema.

In the present study, we aimed to investigate the effect of macular blood flow on the CT and retinal thickness using OCTA. Using SS-OCTA, we found that the CT decreased significantly and was associated with the ultrafiltration volume after haemodialysis; however, the retinal thickness did not undergo a significant change. Similarly, the OCTA results indicated that the decrease in the perfused vessel density occurred at the level of the choriocapillaris; there was no change in the perfused vessel density at the level of the SCP and DCP. Only one OCTA study by Zhang *et al*. investigated the changes in the retina and choroid after haemodialysis^21^. They reported that the retinal thickness and vessel density of the outer retina were significantly decreased but the CT and vessel density of the SCP, DCP, and choriocapillaris layer were not after haemodialysis. These findings differed from those of our study, perhaps due to differences in the OCTA device (SD-OCTA vs. SS-OCTA) and protocol differences (3 × 3 mm vs. 6 × 6 mm)^21^. We did not evaluate the vessel density of the outer retina because, if there was no retinal pathology, no visible flow was observed on OCTA in the outer retina^30^. Further OCTA studies are warranted to confirm the effect of haemodialysis on the retinal and choroidal vasculature.

In order to interpret our results, we reviewed previously published papers. Rubinger *et al*. reviewed 20 previous studies and reported an increase in sympathetic activation after haemodialysis. They commented that adequate sympathetic activation is an essential compensatory mechanism for the maintenance of BP during haemodialysis^31^. Sander *et al*.^32^ reported that sympathetic activation of the choroidal smooth muscles induces vasoconstriction and can cause a decrease in the CT. The change in perfused vessel density after haemodialysis can be explained by the choroidal and retinal characteristics. During haemodialysis, ultrafiltration leads to blood volume depletion, which is associated with disturbed retrobulbar circulation^1–5^. Numerous studies have demonstrated that the retina has an autoregulatory mechanism wherein a moderate change in perfusion pressure has a negligible influence on retinal blood flow^33,34^. In contrast, the choroidal circulation lacks autoregulation; it exhibits autonomic regulation with changes in blood flow directly related to perfusion pressure. These mechanisms are consistent with our results that indicated a change in the perfused vessel density of the macula after haemodialysis. Therefore, it can be assumed that the decrease in the CT and choriocapillaris perfused vessel density after haemodialysis occurs due to the fluid volume depletion itself or due to the sympathetic vasoconstriction of the choroidal vessels caused by loss of fluid during haemodialysis.

After haemodialysis, the perfused vessel density at the choriocapillaris level and the CT decreased to a similar extent, but these changes did not exhibit a significant correlation in this study. Instead, the decrease in the choriocapillaris perfused vessel density was associated with a decrease in BP, including SBP and MAP. However, there was no correlation between the changes in BP and the ultrafiltration volume associated with the CT. The factors associated with the changes in the choriocapillaris perfused vessel density and CT are neither constant nor correlated with each other. This is presumed to be due to the dynamic known and unknown systemic compensatory mechanisms involved in the maintenance of homeostasis during haemodialysis. Further studies are warranted to clarify these unidentified mechanisms affecting the choroid during haemodialysis.

In this study, there were no differences between patients with and without DM in any parameters. Celikay *et al*.^35^ reported that the decrease in the CT after haemodialysis did not differ between patients with and without DM. However, Chang *et al*.^26^ reported that haemodialysis has a greater effect on the CT in patients with DM than in patients without DM, suggesting that the histological and autonomic nervous system changes that occur in diabetes are affected by haemodialysis.. In our study, the patients with DM had NPDR, and our findings should be interpreted in this context. Further studies are necessary to investigate the effect of haemodialysis according to the grade of diabetic retinopathy.

Our study has several strengths. First, this study differs from previous studies; we used SS-OCTA for the first time to measure the effect of haemodialysis on the macula. Unlike conventional imaging modalities such as FFA, ICGA, and USG, the perfused vessel density of each layer was measured quantitatively by auto segmentation of SS-OCTA. Second, we used the ETDRS grid using the 6 × 6 mm protocol. Unlike the focal evaluation of the retina and choroid in previous studies, we were able to assess the whole macular area of the choroid and retina and evaluate the relationship between them in each subfield. Third, our measurements were performed immediately after the first haemodialysis session of the week with longer breaks over the weekend; hence, the effect of haemodialysis on the choroid and retina could be maximised, and the effects of homeostatic mechanisms could be minimised.

However, this study has some limitations. First, one of the major limitations was the heterogeneity of enrolled participants with variable aetiologies of ESRD. In addition, the sample size was relatively small. The retrospective power calculation of the current study was approximately 39%. A prospective study with a larger sample is warranted to obtain greater statistical power. Second, it should be noted that there are inevitable limitations of the current OCTA technology. High-resolution volumetric blood flow images of OCTA based on motion contrast imaging of erythrocytes is limited with regard to the choriocapillaris. Although the choroid has the highest blood flow per unit of tissue weight in the human body, OCTA can miss areas of slower blood flow under a minimum threshold^30^. Additionally, most commercially available OCTA systems display motion and projection artefacts^30,36^. Although most OCTA platforms have in-built software to reduce the projection artefacts, and novel algorithms to remove artefacts are currently being developed, there is no “gold standard” method to address this problem in OCTA evaluation^24,37–39^. In this study, we used the SS-OCTA instrument with the longest wavelength of the commercially available OCTA and a real-time tracker that actively follows the fixation movements. A recent report indicated that the SS-OCTA used in this study resulted in fewer projection artefacts than other OCTA based on SD-OCT because of its swept-source light source^40^. We also aimed to minimise artefact-related variation by performing a paired comparison of images taken by the same photographer, in the same clinical setting, at an approximate 4 h interval immediately before and after haemodialysis. Despite these efforts, we could not fully remove the projection artefacts observed in the DCP and choriocapillaris layers. However, we found that, in the same enrolled eye, the same projection artefacts were presented on OCTA images before and after haemodialysis. As we obtained paired perfused vessel density data of the same eye of the same patient before and after haemodialysis, the projection artefacts can be minimised by subtracting the vessels causing artefacts by paired comparison. The value of the vessel density induced by the projection artefact can also be offset, although it was necessary to assume that the vessels causing the artefacts did not undergo any change after haemodialysis. Tow *et al*. demonstrated that the central retinal venules were maximally dilated immediately after haemodialysis by measuring the retinal vascular calibre on the retinal fundus photograph^41^. These changes may influence the results of our study. However, we did investigate capillary-sized retinal vessels rather than major retinal vessels. In our study, the perfused vessel density in the outer superior and inferior subfields was significantly different after haemodialysis. In the outer superior subfield, the perfused vessel densities of the SCP and DCP were decreased, while in the outer inferior subfield, the perfused vessel densities were increased. This could indicate that, in the outer superior subfield, a decrease in the perfused vessel density in the choriocapillaris layer may be associated with bias including a decrease in the vessel density in the SCP and DCP by the projection artefacts. However, our results revealed a decrease in the vessel density in the choriocapillaris layer after haemodialysis in the outer inferior subfield. This finding suggests that the changes in the retinal capillary vessels were not significant, that the projection artefacts were not affected, or that the decrease in the vessel density in the choriocapillaris was large enough to offset the effects of the SCP and DCP changes. Therefore, almost all subfields aside from the outer superior area could be meaningful with consistent reductions in the perfused vessel density of the choriocapillaris after haemodialysis. In addition, most projection artefacts were observed at the outer ring of the ETDRS grid. There were no vessels in the central area (foveal avascular zone); therefore, the vessel density of the central subfield area in the choriocapillaris layer was not affected by the projection artefacts. Third, our study was performed on patients who underwent haemodialysis after a two-day break over the weekend. This may have exaggerated our results compared to those of haemodialysis conducted during the week with only a one day break. Thus, our results should be interpreted with caution. Future studies with improved technology and a larger sample size are required.

Although there were several limitations to our study, this is the first study to measure the changes in the macula after haemodialysis using SS-OCTA. We found that the choriocapillaris perfused vessel density measured using SS-OCTA decreased after haemodialysis, whereas the perfused vessel density of the SCP and DCP was unaffected. However, further studies are warranted to confirm the mechanism underlying the changes in the choriocapillaris perfused vessel density and the CT after haemodialysis.

Our study demonstrated that evaluation of the vessel density in the choroidal layer using SS-OCTA may serve as a window to assess the systemic perfusion status. In the future, with further studies on the relationship among the retinal, choroidal, and systemic blood vessels, OCTA may become an effective non-invasive tool with which to identify the macular changes associated with systemic vascular diseases.
